# The elusive clinical significance of osteocalcin actions in energy metabolism in humans

**DOI:** 10.20945/2359-3997000000050

**Published:** 2018-05-07

**Authors:** Bruno Ferraz-de-Souza

**Affiliations:** 1 Universidade de São Paulo Hospital das Clínicas Faculdade de Medicina Universidade de São Paulo São Paulo SP Brasil Laboratório de Endocrinologia Celular e Molecular LIM-25 e Unidade de Doenças Osteometabólicas, Divisão de Endocrinologia, Hospital das Clínicas da Faculdade de Medicina da Universidade de São Paulo (HCFMUSP), São Paulo, SP, Brasil

In spite of its stagnant gross appearance, the skeleton is a highly metabolically active organ. The continuous process of bone remodeling, allowing homeostatic availability of calcium and renovation of the important structural material of bone, requires ample energy supply (1). Extreme examples of such energy demand had been noticed a long time ago in high bone turnover states, for instance in the cachexia accompanying severe hyperparathyroidism or in slender children with osteogenesis imperfecta, but lacked an explanation. Therefore, when the first mechanistic pieces of evidence linking bone and energy metabolism in animal models started arising (2), it not only made sense in physiological terms, but it was also felt as if by identifying these missing links we would potentially be able to tackle simultaneously two major health care concerns, adiposity and osteoporosis.

It has now been more than 10 years since the paramount work of Lee and cols. placed osteocalcin (Oc) as the foremost bone-derived hormone to influence energy metabolism (3), rocketing Oc from its role as a (perhaps second rate) bone formation marker to a means by which bone, as an endocrine gland, would direct insulin sensitivity and production, and fat accumulation. From then onwards, subsequent evidence have solidified that, in mice, Oc favors glucose tolerance and insulin sensitivity (4,5). Its role as an energy metabolism effector in humans, however, remains elusive.

Osteocalcin is produced by osteoblasts and secreted in a fully carboxylated form into the bone matrix, where it acts to regulate bone mineralization. Under acidic pH in the osteclastic resorption lacuna, it suffers decarboxylation generating undercarboxylated Oc – importantly, this is the form of Oc that has been chiefly linked to a metabolic role, but its determination in serum is not straightforward in clinical or even in research settings (5). Several studies in humans have investigated the relationship of total serum Oc and a variety of metabolic parameters, and results have been conflicting (6). Indeed, an effect, when seen, was considerably smaller in magnitude to what was observed in mice.

Two articles in this issue of the *Archives of Endocrinology and Metabolism* (AE&M) have aimed to shed light on this topic through different approaches. Campos and cols. have analyzed clinical, laboratory and anthropometric and body composition parameters in a cohort of 34 obese adolescents submitted to a one-year interdisciplinary intervention including exercise, diet and psychological support (7). As expected, the intervention resulted in a reduction in body mass and fat was seen, accompanied by a significant increase in adiponectin and in lean mass. Even though an increase in bone mineral content was observed after the 1-year intervention, the expected progression of bone accretion during this period of life precludes ascertaining a positive effect of exercise on bone formation. The authors focused their analysis on the relation between adipokines and bone turnover markers, finding a positive correlation between the adiponectin/leptin ratio (a marker of adipose tissue dysfunction and insulin resistance) and osteocalcin at baseline. While it is tempting to suggest that the metabolic improvement in insulin resistance and adiposity that resulted from their concerted intervention efforts led to bone formation, it should be noted that at the end of the intervention period, the observed (and expected) increase in the adiponectin/leptin ratio was not accompanied by significant changes in Oc.

Barroso and cols. report a cross-sectional analysis of a large longitudinal birth cohort in Ribeirao Preto consisting of 468 young adults, evaluating bone and metabolic parameters (8). The mean age of subjects was 23 years, meaning that peak bone mass might had not been attained by most participants and that the physiologic scale could still be tipped in favor of bone formation. The majority of the cohort had normal body mass index and waist circumference, and moderate to high physical activity, showing that these were mainly metabolically healthy subjects. While positive findings included a correlation between bone mineral density and HOMA-estimated glucose metabolism, that was modified by visceral adiposity as assessed by waist circumference, no significant association between osteocalcin and glucose metabolism was seen.

Collectively, these two papers exemplify the frustrating quest for the role of osteocalcin in human energy metabolism. Notably, both papers analyzed total osteocalcin, demonstrating the persisting difficulties of regularly studying undercarboxylated Oc; it could be speculated that results might have been different if the more “metabolically active” form of Oc had been analyzed. However, the bulk of the literature by now points towards confusion in this matter, and it is unlikely that further association studies will help elucidate it. In fact, osteoporosis treatment with antiresorptive agents for the past decades has effectively manipulated levels of Oc in hundreds of thousands of individuals, and a striking deleterious effect in energy metabolism has not been seen. As human Oc is yet unavailable as a drug to be tested in randomized controlled intervention trials, the question remains whether, in humans, circulating osteocalcin is physiologically relevant as a marker or an effector of metabolic health.
